# A bioinformatics approach to identify a disulfidptosis-related gene signature for prognostic implication in colon adenocarcinoma

**DOI:** 10.1038/s41598-023-39563-y

**Published:** 2023-07-31

**Authors:** Gunchu Hu, Hongliang Yao, Zuxing Wei, Linye Li, Zhuowen Yu, Jian Li, Xiong Luo, Zhushu Guo

**Affiliations:** 1 Department of General Surgery, The Second Xiangya Hospital of Central South University, Changsha, 410011 China; 2 Clinical Nursing Teaching and Research Section, The Second Xiangya Hospital Central South University, Changsha, 410011 China; 3 Department of Orthopedics, The Second Xiangya Hospital of Central South University, Changsha, 410011 China; 4 Hunan Key Laboratory of Tumor Models and Individualized Medicine of Hunan Province, Changsha, 410011 China

**Keywords:** Bioinformatics, Genomic analysis

## Abstract

Colon adenocarcinoma (COAD) is a type of cancer that arises from the glandular epithelial cells that produce mucus in the colon. COAD is influenced by various factors, including genetics, environment and lifestyle. The outcome of COAD is determined by the tumor stage, location, molecular characteristics and treatment. Disulfidptosis is a new mode of cell death that may affect cancer development. We discovered genes associated with disulfidptosis in colon adenocarcinoma and proposed them as novel biomarkers and therapeutic targets for COAD. We analyzed the mRNA expression data and clinical information of COAD patients from The Cancer Genome Atlas (TCGA) database and Xena databases, extracted disulfidptosis-related genes from the latest reports on disulfidptosis. We used machine learning to select key features and build a signature and validated the risk model using data from the Gene Expression Omnibus (GEO) database and Human Protein Atlas (HPA). We also explored the potential biological functions and therapeutic implications of the disulfidptosis-related genes using CIBERSORTx and GDSC2 databases. We identified four disulfidptosis-related genes: TRIP6, OXSM, MYH3 and MYH4. These genes predicted COAD patient survival and modulated the tumor microenvironment, drug sensitivity and immune microenvironment. Our study reveals the importance of disulfidptosis-related genes for COAD prognosis and therapy. Immune infiltration and drug susceptibility results provide important clues for finding new personalized treatment options for COAD. These findings may facilitate personalized cancer treatment.

## Introduction

Colon adenocarcinoma (COAD) is a type of cancer that affects the cells that line the colon. It accounts for most cases of colon cancer^1^. The best treatment for COAD depends on how advanced the cancer is, how healthy the patient is and what they prefer. COAD can be cured with surgery and other treatments if it is found early^2^. But it can come back or resist drugs and make treatment harder^3^. COAD has become more common and deadly cancer in China in recent years^4–6^. Studying the molecular features of COAD can help doctors predict how it will behave and choose the best treatment for each patient^7^. It is important to educate people about COAD and how to prevent it.

Disulfidptosis is a new way of cell death that happens when cells have too many disulfide bonds in their proteins^8^. Disulfide bonds are links that hold proteins together, but too many of them can make proteins work poorly or break down. Disulfidptosis is caused by low glucose in cells that make a lot of SLC7A11, a protein that helps cells get cystine and make glutathione^9^. Cystine and glutathione are important for keeping cells healthy and balanced^10,11^. Low glucose makes SLC7A11 stop working and mess up the cell balance, causing oxidative stress and more disulfide bonds^12,13^. Disulfidptosis mainly affects the actin cytoskeleton, which is a structure made of protein threads that gives cells shape and strength^14,15^. Disulfide bonds make the actin threads fall apart and clump together, killing the cell. Disulfidptosis is different from other types of cell death because it involves disulfide proteins and actin collapse. Disulfidptosis can influence cancer prognosis and treatment efficacy by modulating tumor microenvironment, drug sensitivity and immune microenvironment.

Prognostic biomarkers are indicators of the future outcome of a disease, such as survival, recurrence, or treatment response. They include genetic, epigenetic, transcriptomic, proteomic, and imaging features. However, many existing biomarkers are inaccurate, inconsistent, non-standardized, and poorly validated in clinics. Therefore, more research is needed to find and verify new and reliable biomarkers for different diseases and populations. Our study focuses on biomarkers that reflect the overall cancer outcome regardless of therapy. They can help diagnose and monitor tumors, and guide personalized treatment strategies.

We got the COAD sample data from the TCGA database. We then used previous studies to find genes related to disulfidptosis. We built and tested a model to predict these genes in COAD using the GEO database. We also looked at immune cells and drug responses in COAD. The goal of this study is to make prognostic markers for COAD and explore how they work and what they mean for COAD patients. We also want to suggest new ways to treat COAD.

## Materials and methods

### Data collection and preparation

We collected and prepared the data as follows. We downloaded the mRNA-sequencing data and clinical information of 426 COAD patients from The Cancer Genome Atlas Program (TCGA) database (https://www.cancer.gov/about-nci/organization/ccg/research/structural-genomics/tcga, accessed on 28 February 2023). We extracted mRNA expression data for COAD patients from TCGA using R. The data contained 60,660 genes in total. After averaging and removing duplicates based on gene names,we curated 59,427 genes with expression values in Transcripts Per Kilobase per Million (TPM). We also curated the survival data of the patients, including progression-free survival (PFS), overall survival (OS), T stage, N stage and M stage, from the UCSC Xena (downloaded from: https://xenabrowser.net/datapages/). We only included COAD patients with survival information in this study. Moreover, we obtained the microarray data GSE39582 (GSE39582 is a dataset of gene expression and clinical data for 566 colon cancer and 19 normal samples from France that was used to identify six molecular subtypes of colon cancer) based on the same platform GPL570 and the corresponding clinical information from Gene Expression Omnibus (GEO) database (https://www.ncbi.nlm.nih.gov/geo/, accessed on 1 March 2023). Based on previous literature^8^, we identified potential disulfidptosis-related genes (DRGs). See Supplementary Table S1 for a detailed list of genes.

### Functional enrichment analysis

We used the “org.Hs.eg.db” R package to convert the symbol ID of each DRG to Entrez Gene ID. Then, we used the “cluster-Profiler” R package^16^ to perform Gene Ontology (GO) enrichment analysis and Kyoto Encyclopedia of Genes and Genomes (KEGG) analysis^17,18^ on the DEGs. We used the “ggplot2” and “ComplexHeatmap” packages^19^ to display the results.

### Filter of the four OS-related genes

We used the R package “forestplot” to select all DRGs based on univariate cox analysis in the total dataset. Then, we used the “riskRegression” package to screen 20 potential prognostic genes. Finally, we used multivariate Cox regression to include only 4 genes in the risk signature.

### Different tissue expression and single gene K–M analysis

We used the R package “ggplot2” to plot box plots to compare the expression of DRGs in different datasets. We used the R package “ggsurvplot” to plot the Kaplan–Meier survival curve to compare the OS between high-expression and low-expression groups for each of the four genes. We obtained the immunohistochemistry (IHC) data from two patients in the Human Protein Atlas (HPA) database. HPA analysis is a method of studying how proteins work and where they are in human cells and tissues using data from the Human Protein Atlas database. Supplementary Table S2 shows their basic information.

### Construction and validation of a prognostic disulfidptosis-related gene signature

We randomly selected 300 samples from the 426 COAD samples in the TCGA-COAD dataset as the train cohort. The other 126 samples were the test cohort. Table 1 shows the baseline clinical characteristics of both cohorts. We used the “glmnet”^20^ function in the R package to use the LASSO algorithm to shrink variables and make specific regression coefficients zero. This gave us a clear model. We used tenfold cross-validation to choose the penalty parameter of the prognosis model based on minimum criteria. Then, we calculated the risk score for each patient as follows: risk score = coefficient Σ(Genei) × expression(Genei), where Genei is the expression of genes and coefficient is the coefficient of multivariate Cox regression. We split all samples into low-risk and high-risk groups based on the median risk score of samples from the train set. We used Kaplan–Meier analysis to compare the OS differences between low- and high-risk groups. We used the “timeROC” package to check how accurate our model predictions were. Then, we used the test set to verify how reliable our model was.

**Table 1:** Clinical characteristics of the TCGA-COAD (train set and test set).

	Train set	Test set
Cases	300	126
Age (median, range)	68 (31–90)	70.5 (37–90)
Gender		
Female	138 (46.00%)	59 (46.83%)
Male	162 (54.00%)	67 (53.17%)
T stage		
T1	6 (2.00%)	5 (3.97%)
T2	51 (17.00%)	23 (18.25%)
T3	207 (69.00%)	85 (67.46%)
T4	36 (12.00%)	13 (10.32%)
N stage		
N0	178 (59.33%)	73 (57.94%)
N1	68 (22.67%)	33 (26.19%)
N2	54 (18.00%)	20 (15.87%)
M stage		
M0	256 (85.33%)	109 (86.51%)
M1	44 (14.67%)	17 (13.49%)
Survival status		
Alive	241 (80.33%)	93 (73.81%)
Dead	59 (19.67%)	33 (26.19%)

### Construction and validation of a nomogram

We used the “rms” R package and the “survival” package to build a nomogram to predict OS. We used the “pROC”^21^ package to do time-dependent ROC analysis to check how well nomogram predicted. We used calibration curves to check how close the actual survival rates and predicted survival rates were.

### Independent datasets validation

We used survival curves from the validation set (GSE39582) to check how reliable our group risk assessment model was. We only included those with OS.time ≤ 80 in the validation set. Table 2 shows the main clinical features of GSE39582. We also predicted the 1-, 2-, 3-, and 5-year survival rates of COAD patients from the validation sets.

**Table 2:** Clinical characteristics of the GSE39582 (OS.time ≤ 80).

	GSE39582
Cases	394
Age (median, range)	69 (22–97)
Gender	
Female	170 (43.15%)
Male	224 (56.85%)
T stage	
T1	7 (1.78%)
T2	35 (8.88%)
T3	255 (64.72%)
T4	97 (24.62%)
N stage	
N0	207 (52.54%)
N1	99 (25.13%)
N2	88 (22.34%)
M stage	
M0	337 (85.53%)
M1	57 (14.47%)
MMR status	
pMMR	310 (78.68%)
dMMR	60 (15.23%)
NA	24 (6.09%)
Survival status	
Alive	235 (59.64%)
Dead	159 (40.36%)

### Immune analysis

We split the whole cohort into two groups of high_risk and low_risk based on the average risk signature point. We used Cell-type Identification By Estimating Relative Subsets Of RNA Transcripts (CIBERSORTx) (https://cibersortx.stanford.edu) to analyze how 22 types of immune cells infiltrated the high_risk and low_risk groups. We used the “estimate” R package to do Estima of Stromal and Immune cells in Malignant Tumor tissues using Expression data (ESTIMATE) analysis to measure the stroma and immune scores. We also used the TIMER database^22,23^ for model gene analysis in COAD.

### Drug sensitivity analysis

We used the “OncoPredict”^24^ R package to find susceptibility data in the GDSC2 database and to predict how COAD would respond to drugs in the GDSC2 database. We used Spearman correlation analysis to find drugs related to risk outcomes and used the “ggstatsplot” package to plot the correlation scatterplot.

### Statistical analysis

We used R 4.2.2 for all statistical analyses. We used the Wilcoxon test to compare nonparametric data from two independent samples. We used t-test and one-way ANOVA to analyze parametric data. We considered a p value < ; 0.05 as statistically significant (p-value < 0.05; *p-value < 0.01; ***p-value < 0.001). We used related R packages such as ggplot2, ggpubr, survival, sur-vminer, and others from the Bioconductor package or the R package. We considered a p value < 0.05 as statistically significant unless we said otherwise.

## Results

### GO and KEGG analysis

**Figure 1:**
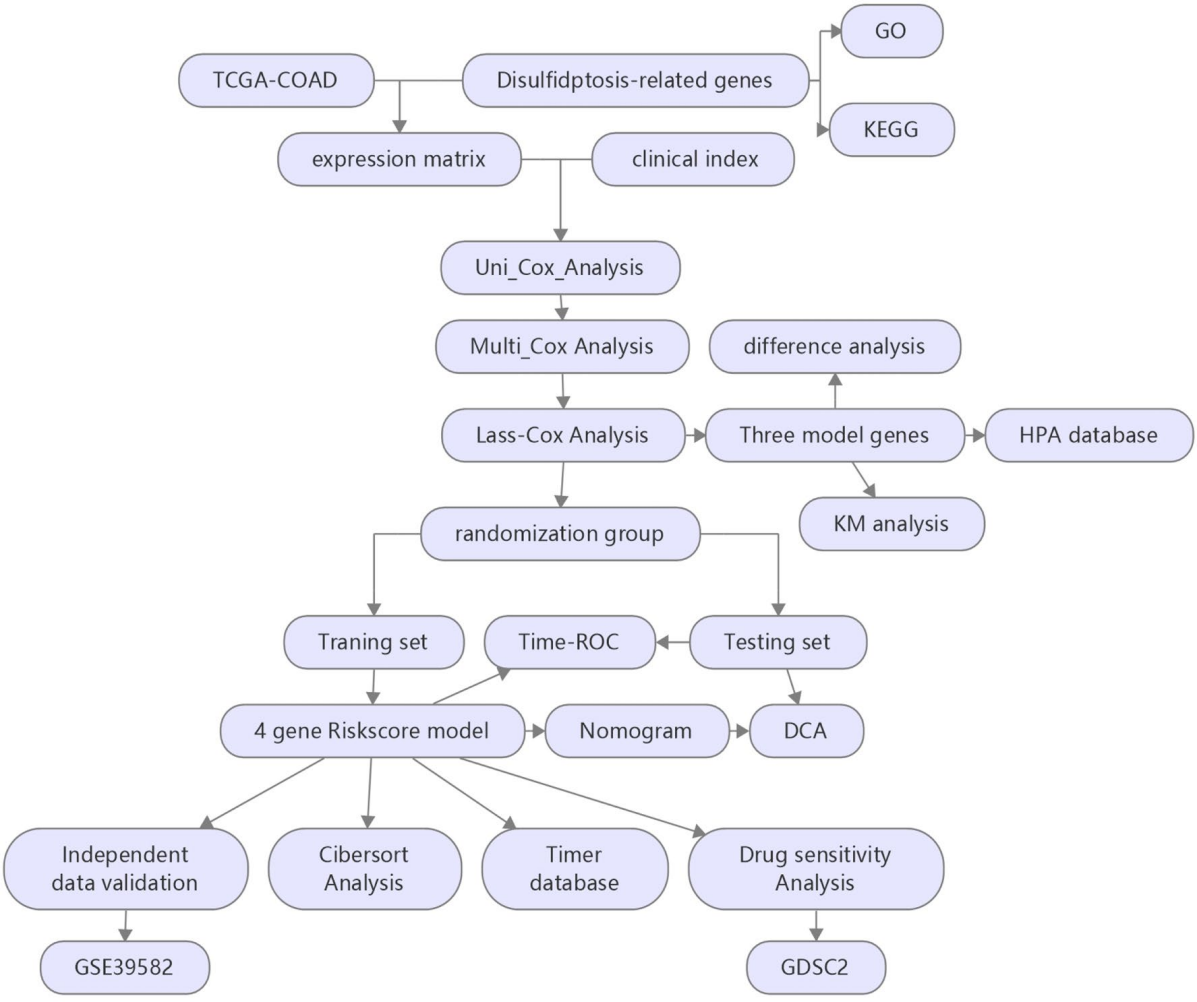
The study exploration workflow.

**Figure 2:**
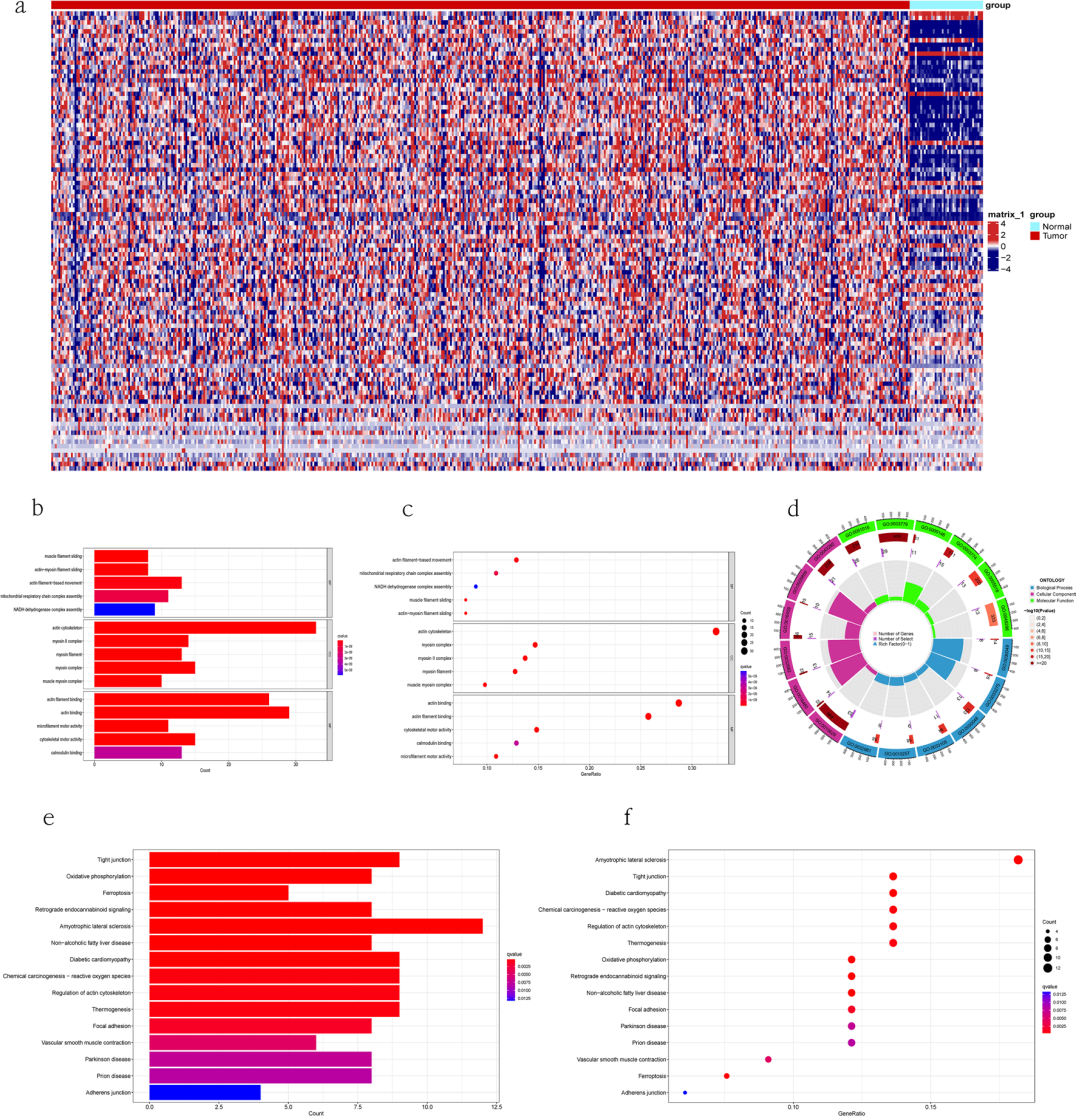
Analysis of dsulfidptosis-related genes with TCGA (**a**) Heatmap of DRGs identified in integrated microarray(tumor in red and normal in blue), (**b**–**d**) Results of GO analysis with DRGs, (**e**,**f**) Results of KEGG analysis with DRGs.

We made a list of 105 genes (Supplementary Table S1) that work closely with Disulfidptosis. Figure 1 shows our study. Figure 2a shows the heatmap of disulfidptosis-related genes in tumor and normal tissues in the TCGA cohort. GO analysis showed that these 107 DRGs were mostly involved in biological processes like muscle filament sliding, actin-myosin filament sliding and actin filament-based movement, cellular components like actin cytoskeleton mysoin II complex and myosin filament, and molecular functions like actin filament binding, actin binding and microfilament motor activity (Fig. 2b–d) (Supplementary Table S2). The KEGG pathway enrichment analysis showed that these DRGs were involved in pathways like amyotrophic lateral sclerosis, tight junction, diabetic cardiomyopathy, chemical carcinogenesis-reactive oxygen species and oxidative phosphorylation (Fig. 2e,f) (Supplementary Table S3).

### Filter disulfidptosis-realted genes

**Figure 3:**
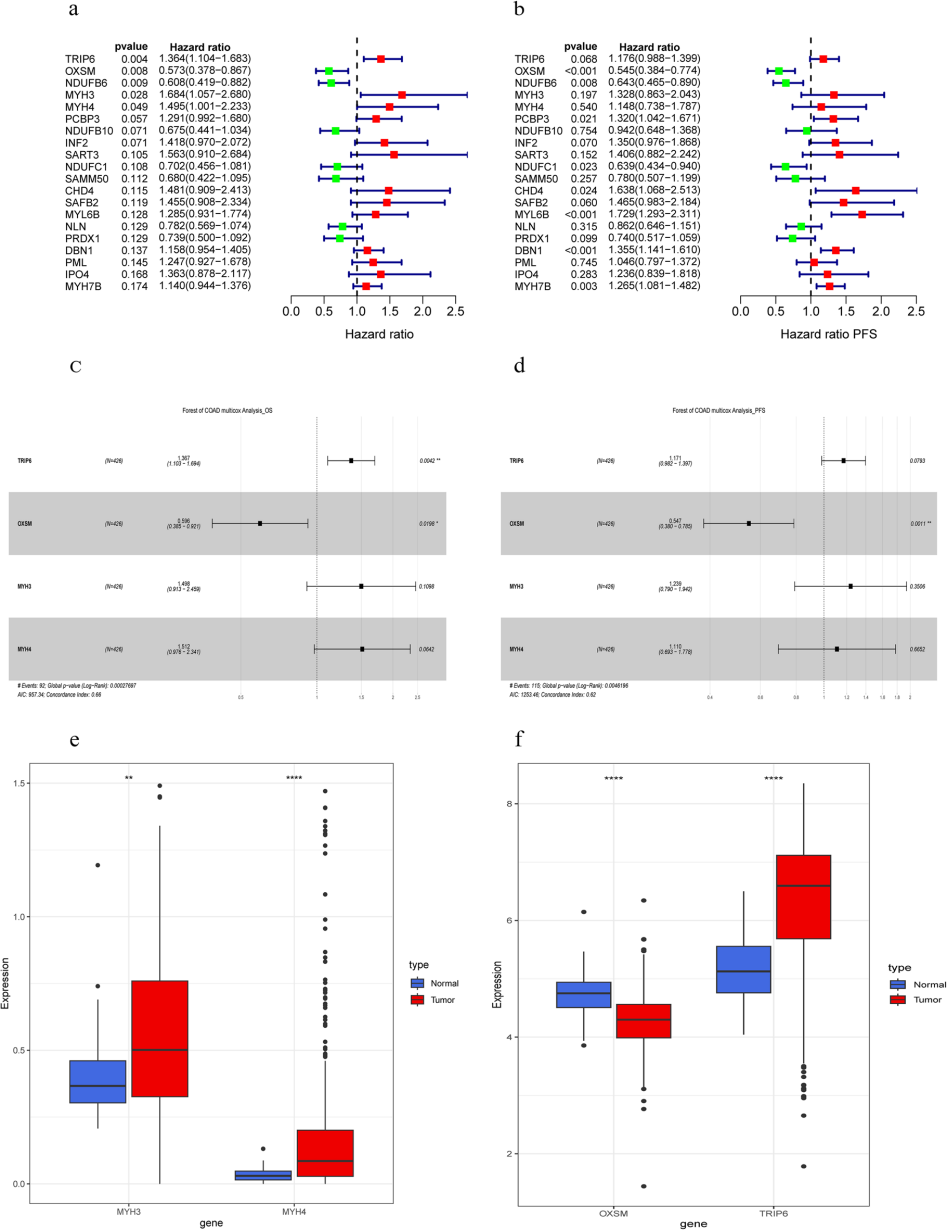
Forest plots DRGs from univariate and multivariate Cox proportional hazards mode. (**a**) The effect of 20 DRGs on the overall survival of TCGA datasets, (**b**) The effect of 20 DRGs on the progress free survival of TCGA datasets, For (**c**) OS and (**d**) PFS, hazard ratios and p-value of the constituents involved in multivariate Cox regression in COAD. (**e**) The expression of MYH3 and MYH4 in COAD and normal tissues (tumor in red and normal in blue). (**f**) The expression of TRIP6 and OXSM in COAD and normal tissues.

We compared the gene expression levels between normal and tumor tissues of COAD patients from TCGA and found that OXSM was significantly downregulated (p = 5.20 × 10^–9^), while TRIP6 (p = 5.00 × 10^–12^), MYH3 (p = 6.70 × 10^–3^), and MYH4 (p = 6.41 × 10^–7^) were upregulated in COAD tissues (Fig. 3e,f). These genes were selected for the forest plot of univariate Cox regression analysis (Fig. 3a,b). Then, we performed stepwise backward regression on the results separately, using two different sets of variables: one screened by univariate Cox (p < 0.05) and another by all-subsets regression (BSR). Four genes were finally chosen for the forest plot of multicox regression analysis after comparing the two methods (Fig. 3c,d).

**Figure 4:**
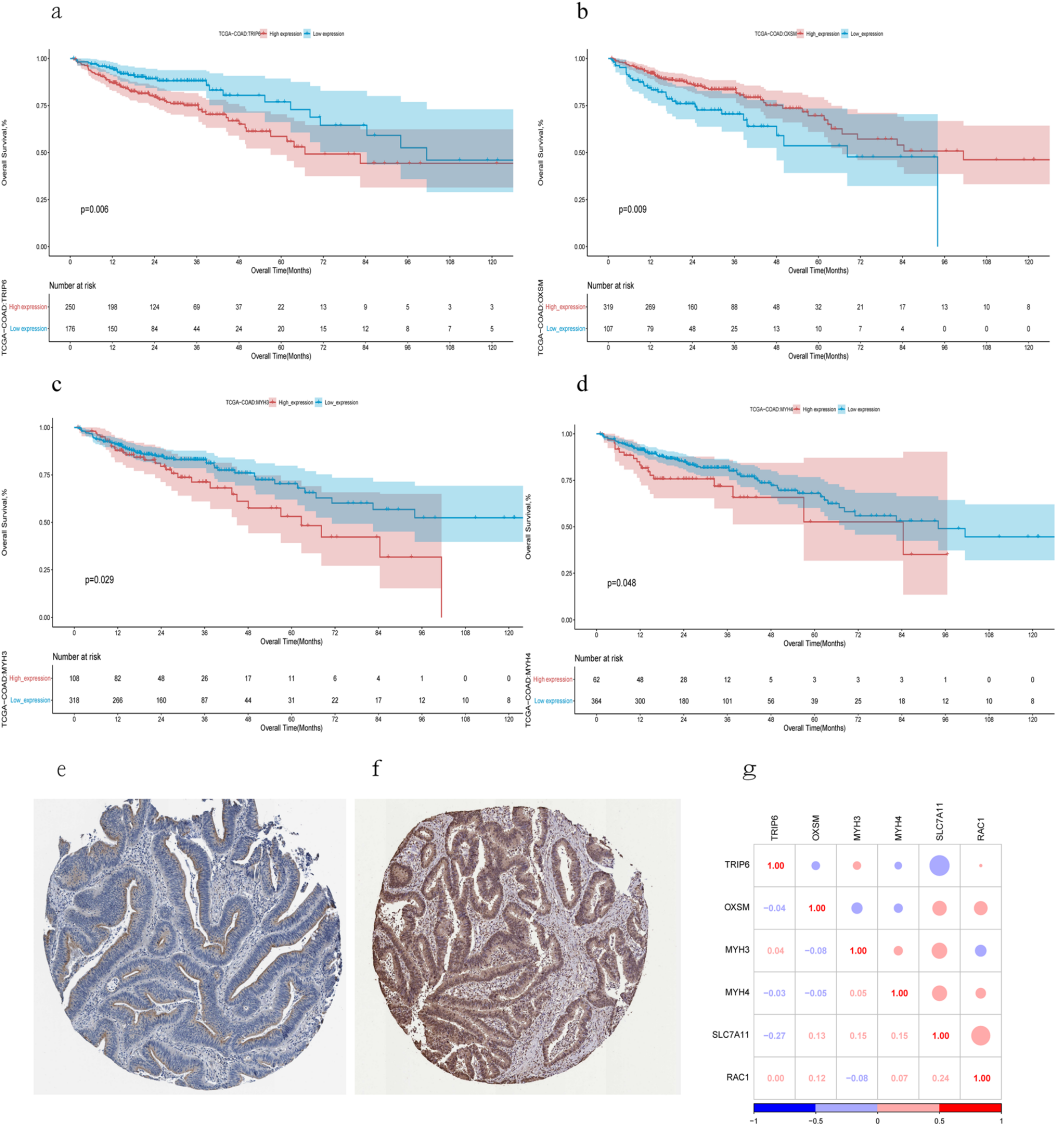
Clinical relevance of DRGs in the COAD patients of TCGA. For OS outcome, Kaplan Meier plot for the expression of (**a**) TRIP6 (**b**) OXSM (**c**) MYH3 and (**d**) MYH4 and overall survival. Representative IHC images of MYH3 (**e**) and TRIP6 (**f**) in COAD tissues. The data was retrieved from HPA database. (**g**) Correlations between the expression of DRGs.

We also assessed the prognostic value of these genes in COAD. All of them were strongly associated with overall survival (OS). The hazards ratios of TRIP6 (HR 1.36 (1.10–1.68), p = 0.006), OXSM (HR 0.57 (0.38–0.87), p = 0.009), MYH3 (HR 1.50 (1.01–2.23), p = 0.029], and MYH4 (HR 0.70 (0.51–0.96), p = 0.048) were statistically significant (Fig. 4a–d). Moreover, we searched the HPA database for immunohistochemical staining data of TRIP6 and MYH3 in tumor tissues and observed higher protein expression of these genes in analyzed tumor tissues (Fig. 4e,f). In addition, we investigated the correlation between the expression of different genes and found some associations (Fig. 4g).

### Establishment of the four-gene risk signature

**Figure 5:**
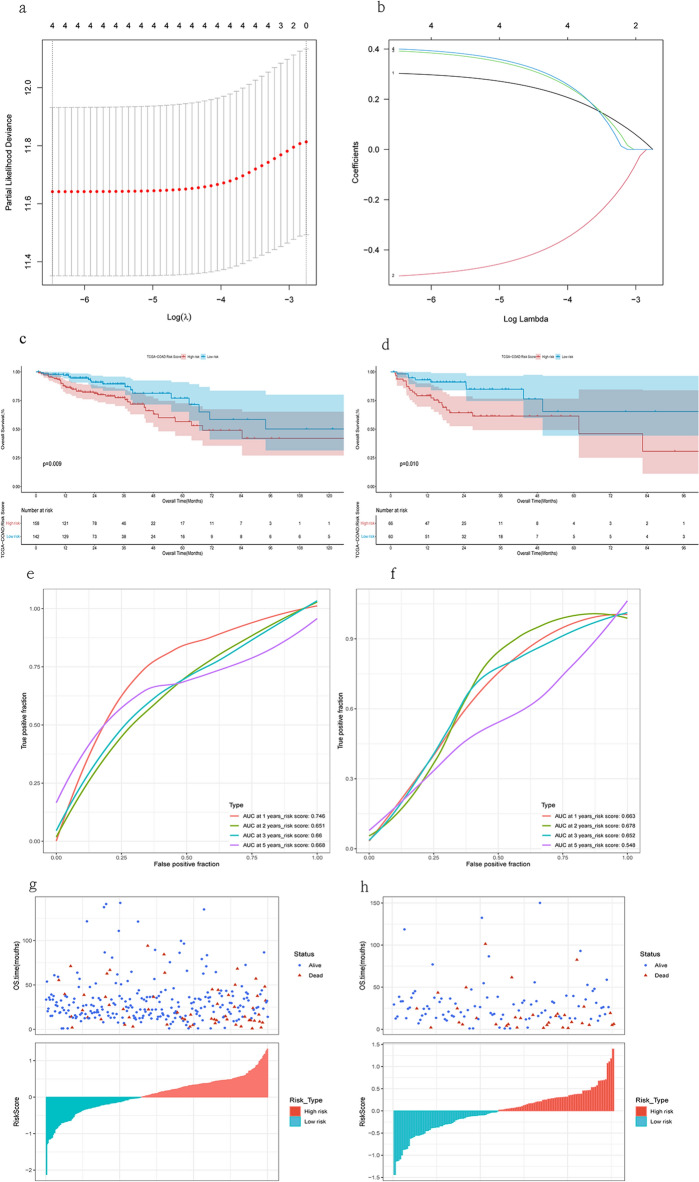
Construction and evaluation of a risk score model. (**a**,**b**) DRGs associated with prognosis were selected using the least absolute shrinkage and selection operator (LASSO) method. Kaplan–Meier plots of the risk score and overall survival were generated for the train set (**c**) and test set (**d**). ROC curves of the risk score were generated for the train set (**e**) and test set (**f**). The distribution of risk scores and overall survival status was shown for the train set (**g**) and test set (**h**).

We divided 426 cases randomly into a train set and a test set (Supplementary Table S6). Table 1 shows their clinical features. We used Lasso algorithm to select four DRGs as candidate genes (Fig. 5a,b). We generated risk scores based on these genes to predict survival and prognosis in COAD patients:

$${\text{Score}}\, = \,0.{3}0{291} \times {\text{TRIP6}} - 0.{5}0{344} \times {\text{OXSM}}\, + \,0.{39184} \times {\text{MYH3}}\, + \,0.{4}00{15} \times {\text{MYH4}}.$$

KM analysis revealed that higher risk scores were associated with poorer overall survival in both the train set (HR 2.75(1.59–4.74), log-rank p = 2.86 × 10^–4^) and the test set (HR 2.91(1.35–6.26), log-rank p = 6.38 × 10^–3^) (Fig. 5c,d, Supplementary Table S7). We assessed the accuracy of the model predictions using the “timeROC” package (Fig. 5e,f). The results confirmed that higher risk scores indicated worse prognosis and survival time (Fig. 5g,h). We obtained clinical information tables and risk scores for the train set (Supplementary Table S8) and the test set (Supplementary Table S9).

### Building and validating a predictive nomogram

**Figure 6:**
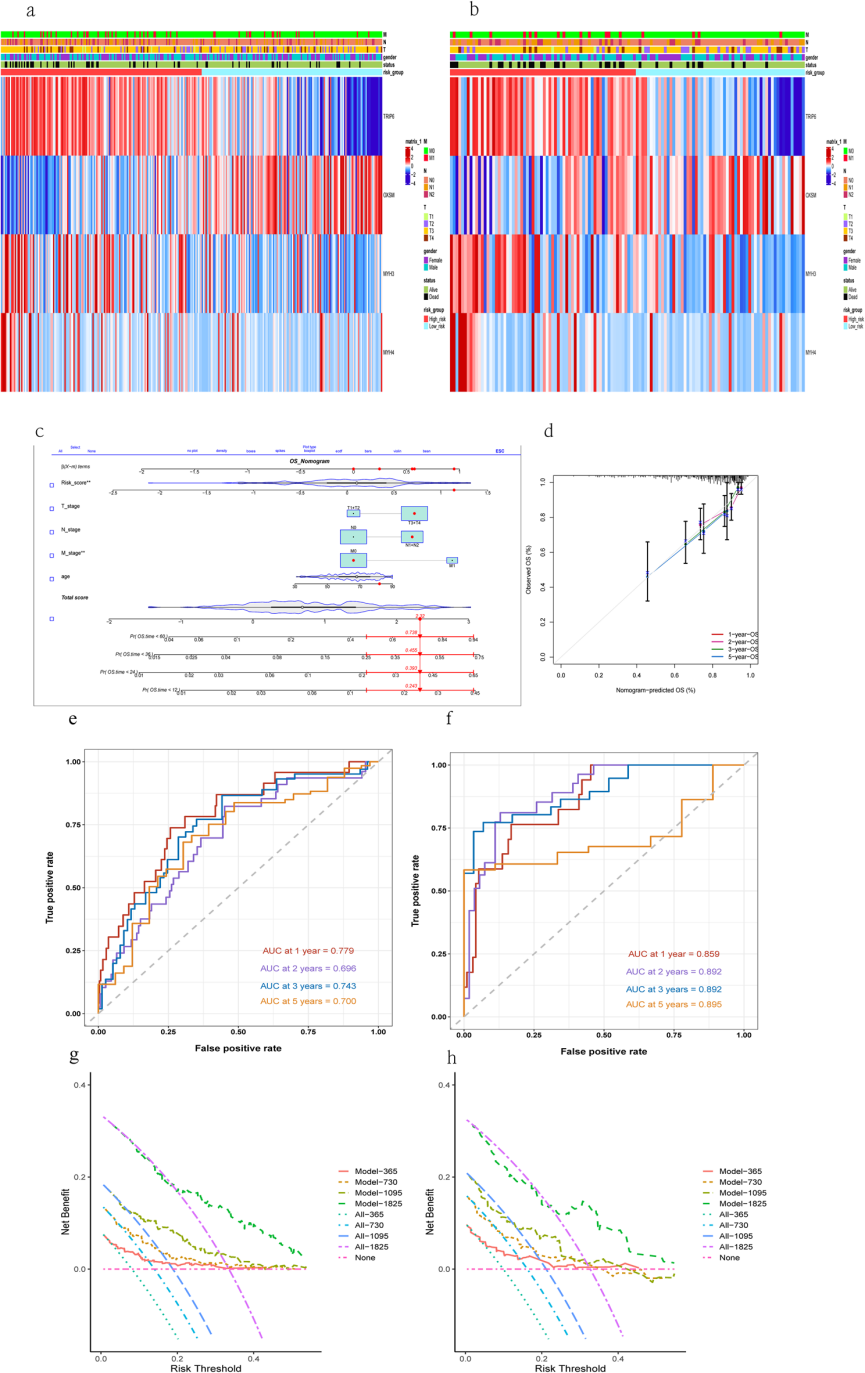
Nomogram development, evaluation and validation. (**a**,**b**) Heatmap of model-genes identified in the train set and test set. (**c**) Nomogram was developed based on the risk scores and different clinical features. (**d**) Calibration plots were constructed to evaluate the predictive performance of overall survival. The ROC curve of the nomogram in the train cohort (**e**) and test cohort (**f**). The decision curves of the nomogram in the train set (**g**) and test set (**h**).

We plotted the expression of model-genes in the train set and test set (Fig. 6a,b). We used risk score, age, T_stage, N_stage and M_stage to build a nomogram for predicting OS (Fig. 6c). The nomogram had a C-index of 0.739 and a good calibration curve (Fig. 6d). The AUCs of the nomogram for 1-year, 2-year, 3-year, and 5-year OS were 0.779, 0.696, 0.743 and 0.700 in train set and 0.859, 0.892, 0.892 and 0.895 in test set (Fig. 6e,f). The decision curves for 1-year OS, 2-year OS, 3-year OS and 5-year OS showed that the nomogram model had a high net benefit for predicting OS of CO-AD patients (Fig. 6g,h).This section should have subheadings to describe the results, their interpretation, and the conclusions.

### Validation of the DRGs signature model based on GEO dataset

**Figure 7:**
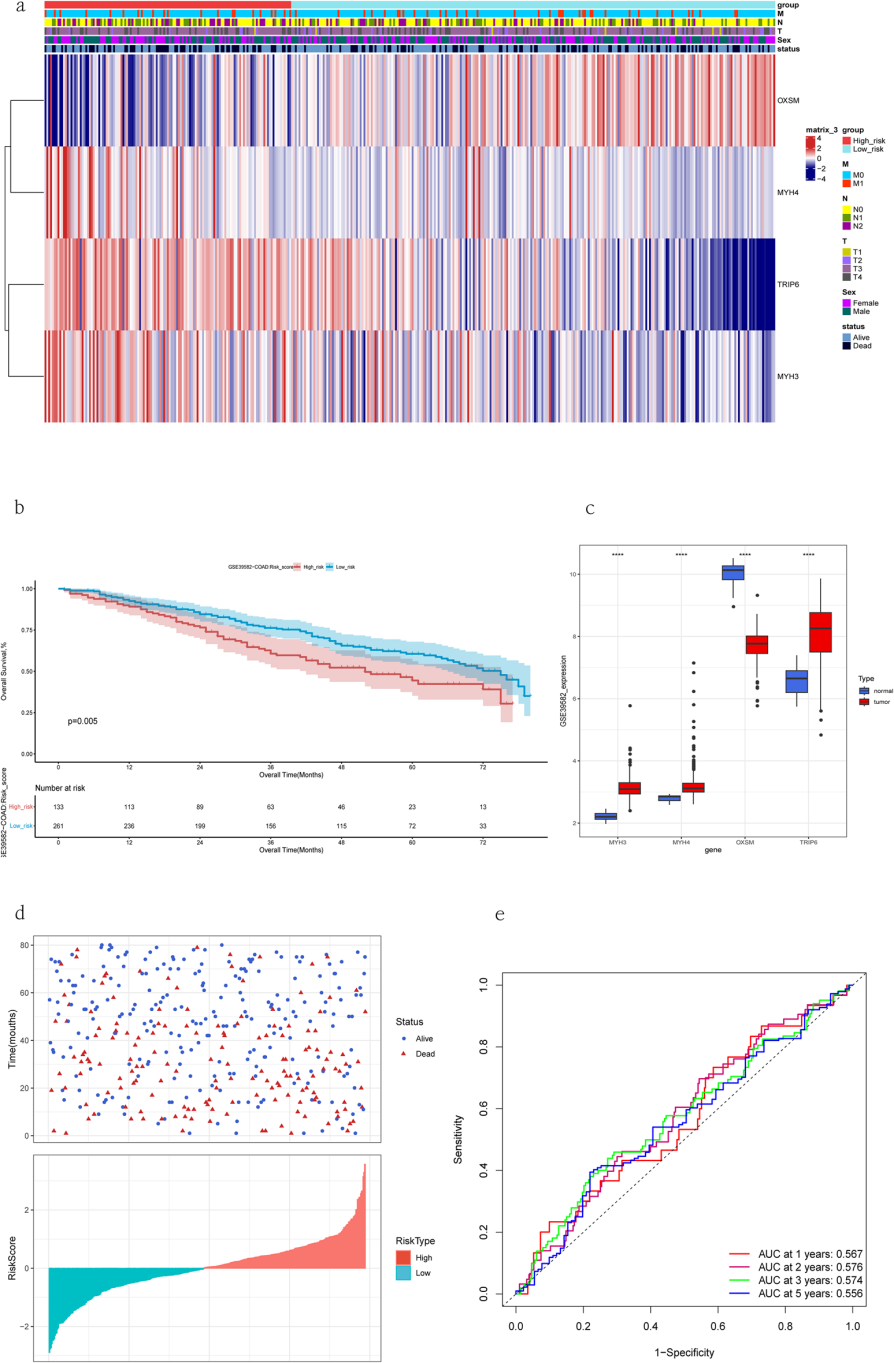
Validation of the seven-gene signature. (**a**) Heatmap of model-genes identified in the validation set. (**b**) Kaplan–Meier plot of risk score and overall survival in the validation set. (**c**) Expression of four genes in COAD and normal tissues in the validation set. (**d**) Distribution of risk score and overall survival status in the validation set. (**e**) ROC curve of risk score in the validation cohort.

We used GSE39582 as the validation data set and selected cases with OS.time ≤ 80 (Table 2, Supplementary Table S10). Figure 7a shows a heatmap of the gene expression distribution of 394 COAD samples. KM analyses indicated that higher risk scores were associated with poorer overall survival in the validation set (Fig. 7b). We compared the expression of model genes in tumor and normal tissues (Fig. 7c). We also displayed the survival status and risk score distributions of these COAD samples using scatterplots and histograms (Fig. 7d). The AUCs for the 1-year, 2-year, 3-year, and 5-year ROC curves were 0.567, 0.576, 0.574, and 0.556, respectively (Fig. 7e).

### Immune analysis

**Figure 8:**
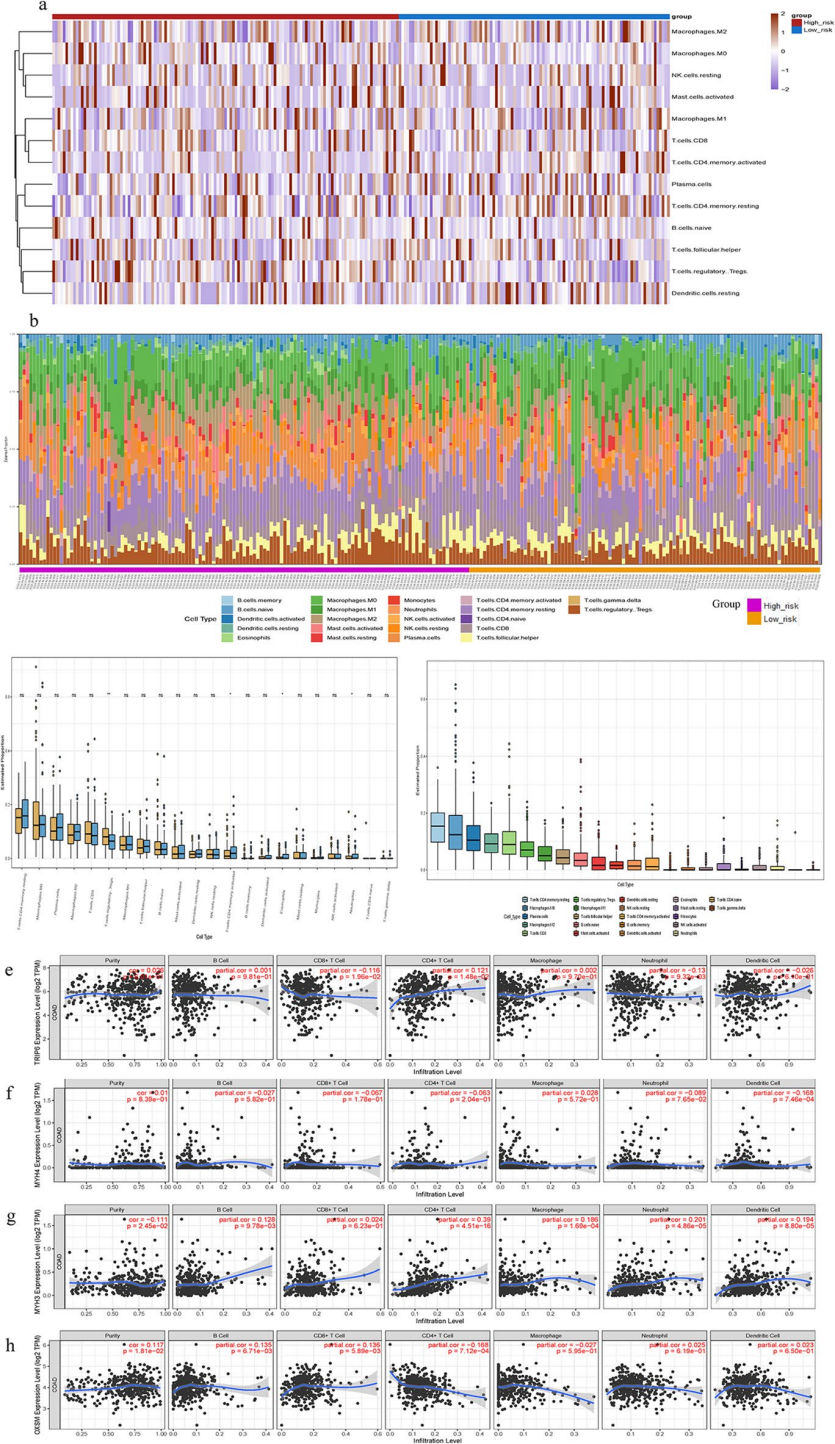
Immune infiltration analysis. (**a**) Heatmap of various immune cell types. (**b**) Infiltration of 22 different immune cell types. (**c**,**d**) Immune cell component between high-risk and low-risk groups. Correlation between (**e**) TRIP6, (**f**) MYH4, (**g**) MYH3, and (**h**) OXSM expression and immune infiltration in COAD in the TIMER database.

To quantify the immune infiltration of tumor samples, we used CIBERSORT, a computational method that estimates the relative abundance of immune cell types from gene expression data. The tumor microenvironment affects COAD occurrence and development, and T cells. Memory CD4 cells, macrophages M0, and plasma cells are the most common tumor-infiltrating immune cells (Fig. 8a,b). We found significant differences in the frequencies of 3 types of immune cells between the favorable and poor prognosis groups (Fig. 8c,d). The ratio of mesenchymal cells to immune cells might differ between the two groups, possibly affecting tumor purity. These results suggest that immune infiltration and immune microenvironment are important for OS in COAD patients.

We investigated whether DRGs would influence immune cell recruitment in the tumor microenvironment and affect COAD prognosis. We examined the relationships between TRIP6, OXSM, MYH3 and MYH4 and immune infiltration in COAD. TRIP6 expression was positively associated with CD4 + T cells (p = 1.48 × 10^–2^) and negatively correlated with Neutrophil (p = 9.32 × 10^–3^) (Fig. 8e). MYH4 expression was negatively correlated with Dendritic cells (p = 7.46 × 10^–4^) (Fig. 8f). MYH3 expression was positively associated with CD4 + T cells (p = 4.51 × 10^–16^),Macrophage (1.69 × 10^–4^) and Neutrophil (p = 4.86 × 10^–5^) (Fig. 8g). OXSM expression was positively associated with Putity (p = 1.81 × 10^–2^), B cells (p = 6.71 × 10^–3^) and CD8 + T Cells (p = 5.89 × 10^–3^) (Fig. 8h).These results suggest that immune parameters can be used as potential biomarkers or therapeutic targets for cancer patients.

### Drug sensitivity

**Figure 9:**
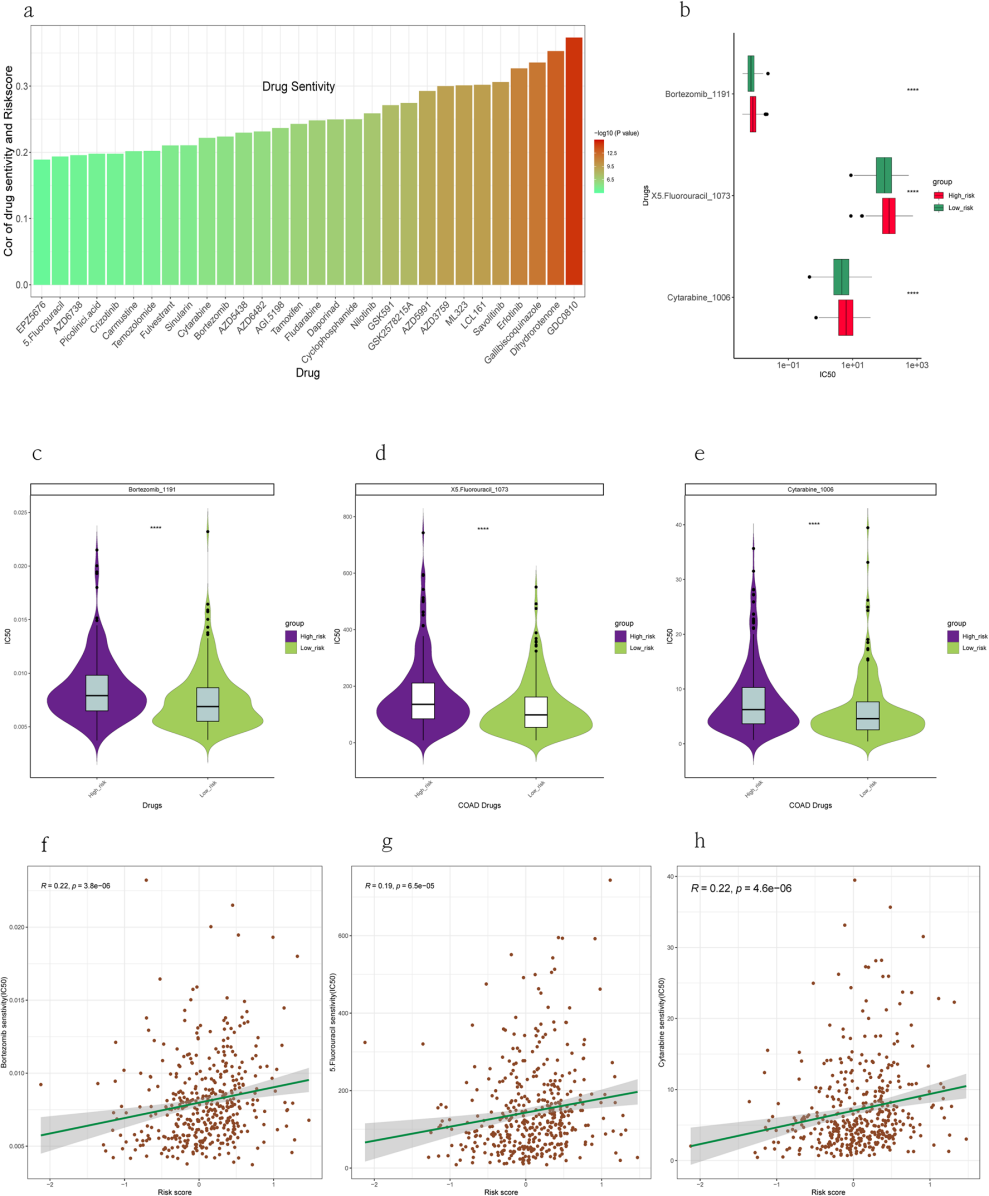
Drug response prediction. (**a**) Comparison of 30 chemotherapy drugs for COAD on the Genomics of Drug Sensitivity in Cancer (GDSC2) database. (**b**) Assessment of drug sensitivity of COAD tumor based on the risk score. (**c**–**e**) Boxplot visualizing different drugs. (**f**–**h**) Correlation analysis between risk score and the IC50 of 3 chemotherapy drugs.

We used the drug sensitivity data from GDSC2 to validate our analytical model and identified some drugs that were associated with drug sensitivity. We assessed the risk scores' value in predicting drug sensitivity in different cancer types. We selected 30 drugs from the GDSC2 database that had significant Spearman correlation between risk scores and drug sensitivity. The risk score was positively correlated with sensitivity to 30 drugs, such as Borte-zomib, 5-Fluorouracil, Cytarabine, Fludarabine, and others (Fig. 9a,b,f–h). The risk score can help us choose appropriate and effective treatment strategies. The IC50 of three drugs was lower in the high-risk group than low-risk group (Fig. 9c–e). These three drugs were Borte-zomib, 5-Fluorouracil, Cytarabine, which could be potential drugs for COAD treatment.

## Discussion

Disulfidptosis happens when cells face disulfide stress, which can be caused by lack of glucose or oxidative stress. Disulfide stress makes disulfide bonds accumulate in actin cytoskeleton proteins, which are important for cell shape and survival. The excess disulfide bonds make actin filaments collapse and aggregate, leading to cell death. Disulfidptosis is different from other cell death types, such as apoptosis, necrosis, autophagy or ferroptosis, because it has a specific target and a specific trigger. Disulfidptosis can be detected by using a fluorescent probe called DCP-Bio1 that binds to disul-fide bonds. DCP-Bio1 can label both inside and outside disulfides and can be used to watch disulfidptosis in live cells or tissues. Disulfidptosis can also be stopped by using antioxidants or reducing agents that stop or undo disulfide bond formation. The finding of disulfidptosis has implications for understanding how cell death and survival work in different normal and abnormal conditions. For example, disulfidptosis may have a role in cancer growth and therapy resistance, as some cancer cells have high levels of disulfide stress and may use disulfidptosis to survive. In fact, Liu et al. did a genetic analysis of 33 kinds of cancers and found that genes related to disulphidesoptosis were often mutated or changed in different cancer types. These genetic changes may affect how cancer cells respond to agents that induce disulphidesoptosis. Therefore, targeting di-sulphidesoptosis may be a potential way to treat cancer. In summary, disulphides are important for controlling cell fate under stress conditions. Disulphides can cause a new form of cell death called disulphidesoptosis by affecting the actin cytoskeleton structure. Disulphidesoptosis has potential implications for various diseases that involve cell death or survival mechanisms. More research on this new pathway may give new insights into the biology of cell death and offer new therapeutic options.

TRIP6 is frequently overexpressed in COAD tissues and cell lines compared to normal tissues that is associated with poor prognosis, advanced tumor stage, lymph node metastasis and distant metastasis in COAD patients^25^. The molecular mechanisms by which TRIP6 promotes COAD tumorigenesis have been investigated by several studies. One study found that TRIP6 can be stabilized by another gene called TTPAL, which is preferentially amplified in COAD^26^. TTPAL prevents TRIP6 from being degraded by the proteasome and enhances its interaction with β-catenin, a key mediator of the Wnt/β-catenin pathway. The Wnt/β-catenin pathway is known to play a crucial role in colorectal cancer initiation and progression by regulating genes involved in cell growth, invasion and stemness. Therefore, TTPAL-TRIP6-β-catenin axis can activate the Wnt/β-catenin pathway and promote COAD. 3-oxoacyl-ACP synthase, mitochondrial (OXSM), also known as FASN2D, is a protein that is involved in the elongation of fatty acid chains in mitochondria^27^. The cis-regulation of enhancers on target genes depends on the action of transcription factors^28^, which are proteins that bind to specific DNA sequences and regulate gene expression. Previous studies^29^ have identified a key transcription factor, core-binding factor subunit beta (CBFB), which is strongly correlated with OXSM expression. In colorectal cancer, CBFB deficiency has been shown to enhance cell resistance to MEK inhibitors^30^, which are drugs that target the mitogen-activated protein kinase (MAPK) pathway. Additionally, hsa-miR-338-3p has been found to participate in the regulation of fatty acid biosynthesis by regulating OXSM levels^31^, and to affect the biological occurrence and rapid proliferation of glioma cells, which are a type of brain tumor.

The instructions for producing myosin-3, a protein that belongs to the myosin family of proteins, which are involved in cellular movement and the transportation of materials within and between cells, are provided by the MYH3 gene. THZ1, a novel covalent CDK7 inhibitor, has been utilized as an anti-tumor drug. It suppresses the expression of differentiation-related transcription factors and muscle structural proteins, including myogenic protein myh3^32^. Encoded by the MYH4 gene, myosin-4 is a protein that belongs to a group of proteins involved in movement and transport within and between cells. It is also expressed in skeletal muscle fibers. A previous study^33^ found that long noncoding RNA Neat1 promoted myoblast proliferation mainly by decreasing the expression of the cyclin-dependent kinase inhibitor P21 gene, but inhibited myoblast differentiation by suppressing the transcription of myogenic marker genes, such as Myh4.

We investigated how disulfidptosis, a type of cell death induced by oxidative stress, affects the genetic and transcriptional landscape of colorectal adenocarcinoma (COAD) and how these changes are linked to each other. We also developed a risk assessment model based on the expression of disulfidptosis-related genes to estimate the prognosis and treatment response of COAD patients. This model can help us reveal the molecular mechanisms and clinical implications of disulfidptosis in COAD and devise more precise and effective immunotherapy strategies. In our experiment, we applied the risk assessment model to assess the immunotherapy response of COAD patients and compared the immune cell composition in tumors with high or low risk scores. CIBERSORT is a computational method that can estimate the relative proportions of immune cell types from bulk RNA sequencing data. It has been widely used to characterize the tumor microenvironment and its impact on cancer biology and treatment. The relationship between disulfidptosis genes and immune cell infiltration is complex and context-dependent. On one hand, disulfidptosis can affect the recruitment and activation of immune cells by modulating the release of damage-associated molecular patterns (DAMPs), cytokines, chemokines, and reactive oxygen species (ROS). On the other hand, immune cells can influence the expression and function of disulfidptosis genes by altering the tumor microenvironment and providing pro- or anti-inflammatory signals. In this study, we found that different immune cell subsets had different correlations with disulfidptosis gene expression and survival outcomes. Our results show that this model can be useful for identifying immunotherapy candidates for COAD patients. GDSC2 is a database that contains drug sensitivity data and genomic features for over 1000 human cancer cell lines. It is a valuable resource for identifying molecular determinants of drug response and developing predictive biomarkers for personalized medicine. The relationship between disulfidptosis genes and drug sensitivity is complex and context-dependent. On one hand, disulfidptosisgenes can affect the response and resistance to drugs by modulating the cellular redox state, protein stability, apoptosis induction, and DNA damage repair. On the other hand, drugs can influence the expression and function of disulfidptosis genes by altering the intracellular or extracellular redox environment, inducing oxidative stress or endoplasmic reticulum stress, or activating or inhibiting signaling pathways. In this study, we found that different drugs had different correlations with disulfide death gene expression and survival outcomes. We also examined the relationship between risk score and drug sensitivity in COAD patients and found that apoptosis, signal trans-duction and metabolism pathways are involved in COAD treatment.

We devised a risk score model with 4 disulfide death genes for survival prediction in COAD. We also uncovered the complex interplay between disulfide death genes and immune cells in the tumor microenvironment and their impact on tumor immunity. Our model has some caveats. It relied on bulk gene expression data, which may not reflect tumor heterogeneity and dynamics. It did not adjust for other confounding factors, such as treatment, comorbidities, or lifestyle. It was based on retrospective data, which may have biases and limitations. Prospective studies are warranted to validate our findings and assess our model's clinical utility. We intend to investigate the mechanisms and functions of disulfide death genes in cancer and therapy. We also aim to target or modulate disulfide death genes as a novel therapeutic strategy for cancer. We aspire to discover better biomarkers based on disulfide death gene expression and other omics data.

## Conclusions

We developed and tested a COAD risk score based on the expression of four genes related to disulfidptosis. This risk score can help us predict the prognosis and immunotherapy response of COAD patients. By analyzing the risk score, we can gain more in-sight into the molecular mechanisms and clinical implications of disulfidptosis in COAD and design more personalized and precise treatment options.

## Supplementary Information

Supplementary Information 1.

Supplementary Information 2.

Supplementary Information 3.

## Data Availability

The data presented in this study are openly available in the TCGA database [https://www.cancer.gov/about-nci/organization/ccg/research/structural-genomics/tcga, (accessed on 28 Feburary 2023)] and GEO database [https://www.ncbi.nlm.nih.gov/geo/, (accessed on 1 March 2023)].
