# Supplementary material for: A bioinformatics approach to identify a disulfidptosis-related gene signature for prognostic implication in colon adenocarcinoma
Source: Sci Rep. 2023 Jul 31;13:12403. doi: 10.1038/s41598-023-39563-y (PMC10390519; doi:10.1038/s41598-023-39563-y)
Supplement: Supplementary file 3 — Supplementary Information 3. [file 41598_2023_39563_MOESM3_ESM.docx]

Supplementary Materials:

Table S1: Disulfidptosis-related genes;

Table S2: The results of GO analysis with DRGs;

Table S3: The results of KEGG analysis with DRGs;

Table S4: As the overall survival outcome, the result of univariate COX regression analysis of DRGs;

Table S5: As the progression-free survival survival outcome, the result of univariate COX regression analysis of DRGs;

Table S6: Comparison results of the total set randomized into train set and test set;

Table S7: The result of calculating the risk ratio of the risk score in three datasets;

Table S8: The overall information table of the train set ;

Table S9: The overall information table of the testing set;

Table S10: The overall information table of the validation set
